# Surgical outcome of repair of aortic valve prolapse and regurgitation associated with ventricular septal defect

**DOI:** 10.12669/pjms.37.3.3067

**Published:** 2021

**Authors:** Tariq Waqar, Muhammad Farhan Ali Rizvi, Jamal Abdul Nasir, Kamran Khan

**Affiliations:** 1 Tariq Waqar, FCPS, FRCS. Associate Professor, Pediatric Cardiac Surgery, Punjab Institute of Cardiology Lahore, Pakistan.; 2 Muhammad Farhan Ali Rizvi, FCPS. Assistant Professor Cardiac Surgery Bahawal Victoria Hospital, Bahawalpur, Pakistan; 3 Jamal Abdul Nasir, FCPS. Associate Professor, Pediatric Cardiac Surgery, Children Hospital Lahore, Pakistan; 4 Kamran Khan, MS. Medical Officer, Punjab Institute of Cardiology Lahore, Pakistan

**Keywords:** VSD closure, Aortic Valve Prolapse, Aortic Regurgitation, Aortic Valve Replacement (AVR)

## Abstract

**Objective:**

To analyze the outcome of repair of aortic valve disease associated with various types of ventricular septal defect.

**Methods:**

In a retrospective observational study design, data of seventy-two patients of ventricular septal defect (VSD) associated with aortic valve prolapse (AVP) and aortic regurgitation (AR) who was operated in Punjab Institute of cardiology from May 2016 to April 2020 was collected. Depending on presence of AR, all patients were divided in four groups. Group-I (VSD and AVP but no AR) had fifteen patients. Only VSD was closed in this group. Group-II (VSD and Mild AR) had forty patients, only VSD was closed in this group as well. Group-III (VSD and Moderate AR) had ten patients, VSD closure and aortic valve repair was done. Group-IV (VSD and severe AR) had seven patients. Aortic valve was repaired in five patients and replaced (AVR) in two patients along with VSD closure. Associated anomalies were addressed as well.

**Results:**

Group-I: Twelve out of fifteen patients (80%) showed no post-operative AR. While two patients (13.3%) showed Trace AR. Single patient (6.6%) showed mild AR. There results were unchanged after mean follow up of 36 months. Group-II: Eight out of forty patients (20%) had no AR, while eight (20%) had trace AR. Twenty-three (57.5%) patients had mild AR. Single (2.5%) patient had moderate AR. After follow up of 24 months the patient with moderate AR progressed to severe AR. We are planning to do Aortic Valve Replacement (AVR) in this case. Rest of cases showed no progression of disease. Group-III: Two out of ten patients (20%) had no AR, four (40%) had trace AR, while four (40%) had mild AR. Mean follow up was 42 months (2.5 years). Neither trace nor mild AR progressed to severe or moderate AR. Group-IV: Among seven patients, five underwent repair while two had AVR. Out of five patients who underwent aortic valve repair, four patients (57.1%) were declared mild AR, while severe AR was converted to moderate AR in single patient (14.28%). Mean follow up was 18 months. The moderate AR patient has progressed to severe AR for last six months and we are planning to do AVR in this patient. Postoperative echo of patients with AVR showed adequately functioning aortic valve with AVPG mean 10 mmHg and 15 mm Hg respectively, with no residual AR.

**Conclusions:**

Aortic regurgitation associated with VSD is a congenital lesion with continuously active aortic valve disease resulting in significant morbidity and mortality. Early diagnosis, effective treatment and meticulous follow up decelerate and in most cases arrest the disease process.

## INTRODUCTION

Pakistan has one of the highest incidences of congenital heart diseases in the world, which is over 9.4/1000 live births.^1^ Ventricular septal defect (VSD) contributes to major share of this complex and challenging situation.^2^ VSD is an enigmatic condition with spontaneous closure rate of 70-85%.^3^ Paradoxically, complications in the undetected and undiagnosed children results in significant mortality and morbidity. Aortic valve prolapses (AVP) and subsequent aortic regurgitation (AR) is a significant complication of undetected VSD, resulting in left ventricular overload, congestive heart failure and ultimate demise in unfortunate patients.^4^ Both perimembranous and doubly committed sub arterial (DCSA) VSDs are associated with AR, with muscular VSD being least associated. There have been some published data quoting incidence of 83% AR associated with DCSA VSD in Pakistani population.^4^ Deficient leaflet support, malformed suspension of commissures, thinned aortic valve cusps and eventually thickened, rigid valvular cusps have been understandably postulated as the pathogenic factors.^5^

In a pilot study, we studied the promising results of repair of aortic valve disease in DCSA VSD.^6^ Nevertheless, the fact is that in our country many VSD cases are undiagnosed till later age and associated AR is usually progressed at much larger stage than reported in western studies.^4^,^6^ A significant proportion even fails to follow up after surgical repair due to lack of comprehensive follow up pathways. Resultantly, published data about extent of aortic valve disease associated with VSD and post-surgical prognosis in Pakistani population is very scarce. Therefore, a comprehensive study encompassing the nature of aortic valve disease associated with all types of VSDs, the immediate outcome of treatment of aortic valve disease along with VSD closure by various surgical techniques and the long-term prognosis of these cases was designed.

## METHODS

Considering a retrospective observational study design, data of 72 patients with VSD who were operated in Punjab Institute of Cardiology (PIC) from May 2016 – April 2020 was collected. Perimembranous VSD were 48 cases; **DCSA VSD** and outlet VSDs 20 and four respectively. VSD without **AVP and AR**, VSDs associated with tetralogy of Fallot (TOF), right ventricle out flow tract (RVOT) obstruction and other complex congenital cardiac diseases were excluded from study. Ethical and academic committee of PIC’s approval was taken (Ref. No: RTPGME-Research-128, Dated: June 02, 2020). Proper consent was taken from all patients after explaining all complications. The patients were referred to us by paediatric cardiologists after diagnosing VSD through transthoracic echocardiography and grading the AR by color Doppler. A pressure half time (PHT) of greater than 500 millisecond (ms) was considered mild AR, 500 ms to 349 ms was moderate AR, 300 ms to 200 ms was moderately severe AR while less than 200 ms was considered severe AR. Patients were divided into four groups on the basis of AVP and degree of AR. Group-I comprised of fifteen patients having VSD along with AVP but no AR. All underwent VSD repair only. Group-II had forty patients of VSD with mild AR, all had VSD repair only as there was mild AR preoperatively, associated anomalies were repaired like closure of patent ductus arteriosus (PDA) in two cases, left ventricle outflow tract( LVOT) resection in one and ruptured sinus of valsalva (RSOV) in two patients. Ten patients were in Group-III having VSD with moderate AR. Associated pathologies were RSOV (1patient), aortic cusp perforation (one patient) and moderate tricuspid regurgitation (one patient). VSD closure and Trusler repair was done in seven cases while aortic cusp augmentation was done in two cases. Right coronary cusp (RCC) augmentation was carried out in one patient and non-coronary cusp (NCC) augmentation in one patient. One patient had repair of aortic cusp perforation. Group-IV had seven cases with VSD and severe AR. Cusp augmentation was done in two cases, new cusp formation with pericardium was done in three patients and AVR was done in two cases. Associated pathologies like RSOV repair and LVOT resection were also performed. Trans thoracic Echo was done before discharge followed by Echo at an interval of 3- and 6-months post procedure and then annually. Statistical analysis was done by SPSS version 23. Quantities variables were presented as mean value and the pre-operative and post-operative quantities variables were compared by ANOVA test. P value of ≤.05 was considered statistically significant; F-ratio was also calculated for comparisons.

### Surgical Technique:

All the patients were operated by standard median sternotomy followed by establishing routine cardiopulmonary bypass. AR was assessed by (1) Echocardiographic findings pre operatively (2) by observing arterial tracing (which shows diminished magnitude of aortic incisura with increasing valve insufficiency) prior to bypass and (3)left ventricular distension on initiation of bypass and while giving cardioplegia. Antegrade cardioplegia was used in cases of mild AR and aortic valve prolapse, while coronary ostial cannula was used for cardioplegia in cases of moderate and severe AR after opening the aorta. Topical cooling with ice and moderate hypothermia were used for myocardial protection. VSD was closed with Dacron patch using pledgetted 5/0 prolene sutures. We commonly used right atrial approach for VSD closure while aortic valve repair was done through aortic approach. DCSA VSDs were closed through pulmonary approach. Trusler repair by suspension of RCC and hood plication of commissure with small pericardial patch was done. Cusp augmentation was carried out for shortened cusps by trimming the RCC and NCC in different cases and augmenting them with glutaraldehyde fixed pericardial patch. New cusp formation was done by excising the deformed cusp. Height of structurally normal cusp especially NCC was used as reference to produce the new cusp with pericardium. Structurally deformed aortic valve (one of which was bicuspid valve) had to be excised in two cases and replaced with mechanical valves of 19 mm St- Jude.

## RESULTS

Mean age of patients was 12.93±6.75 years. There were 18(25%) females and 54(75%) males. Mean weight was 29.04±12.85 kg. Small residual VSD was observed in one patient of Group-I and one patient of Group-II (2.7%), no progression was reported on follow up. Perimembranous VSDs were most common (66%), followed by DCSA (27%) and outlet VSD (5%). No muscular VSD was diagnosed with AR in our study. ICU stay complications i.e.; pneumothorax and chest infections were dealt satisfactorily. Table-I. There was a transient AV block in a single patient which recovered in ICU. There was a single early mortality in a ten year old female patient who underwent DCSA closure and AR repair, she had a stroke in ICU and she expired after seven days in ICU. No late mortalities are reported so far.

**Table-I:** Comparison of Operative Characteristics.

Variables	Group-I (N=15)	Group-II (N=40)	Group-III (N=10)	Group-IV (N=07)	F-Ratio	P-value
Cross-Clamp Time (minutes)	55.86±14.21	54.15±13.50	86.10±32.40	97.85±22.89	17.84	<0.0001
Bypass Time (minutess)	76.93±15.01	83.12±15.10	123.90±41.55	149.28±24.23	28.73	<0.0001
Mechanical Ventilation (hours)	5.6±0.98	5.77±1.38	6.5±1.71	7.71±4.02	2.92	0.04
ICU Stay (hours)	18.53±4.82	22.42±4.37	22.20±6.05	26.57±9.71	3.85	0.013
Hospital Stay (days)	5.53±0.63	5.65±0.83	5.90±0.73	6.42±0.97	2.36	0.078

### Group-I:

Twelve out of fifteen patients (80%) showed no post-operative AR, while two patients (13.3) had trace AR and one patient (6.7%) showed mild AR. The results were unchanged after mean follow up of 36 months.

### Group-II:

Out of forty patients, twenty-three (57.5%) had mild AR, eight patients (20%) had no AR and further eight (20%) had trace AR. One (2.5%) patient had moderate AR. Mean follow up of this group was two years. The patient with moderate AR showed progression to severe AR. VSD type in this case was perimembranous. We have planned for AVR in this case. Rest of cases showed no progression of disease.

### Group-III:

Four (40%) patients had mild AR, another four (40%) had trace AR and two out of ten patients (20%) had no AR. Mean follow up was 2.5 years in this group. Neither trace nor mild AR progressed to severe or moderate AR.

### Group-IV:

Four patients (57.1%) revealed mild AR, two (28.5%) had no AR and one patient (14.3%) was found to have moderate AR. Mean follow up was 18 months in this group. Patient with moderate AR had augmentation of RCC cusp which progressed to severe AR during last 6 months follow up so we have planned for AVR in this patient. Again, the VSD type was perimembranous. Moreover, both the patients with AVR have shown adequately functioning aortic valve with AVPG mean 10 mm Hg and 15 mmHg respectively Table-II.

**Table-II:** Comparison of Pre-Surgery and Post-Surgery Aortic Regurgitation.

		Pre-Op	Post-Op	P-value
	None	15 (100%)	12 (80.0%)	
Group-I	Trace	0	02 (13.3%)	0.18
	Mild	0	01 (6.7%)	
	None	0	08 (20%)	
	Trace	0	08 (20%)	
Group-II	Mild	40 (100%)	23 (57.5%)	0.0001
	Moderate	0	01 (2.5%)	
	None	0	2 (20%)	
	Trace	0	4 (40%)	
Group-III	Mild	0	4 (40%)	0.0002
	Moderate	10 (100%)	0	
	None	0	02 (28.5%)	
	Trace	0	0.0	
Group-IV	Mild	0	04 (57.1%)	0.002
	Moderate	0	01 (14.3%)	
	Severe	7 (100%)	0.0	

## DISCUSSION

VSD is the most prevailing congenital cardiac lesion^7^, with perimembranous VSD as most common subtype.^4^ Although we only selected VSD cases associated with aortic valve problems for this study, still perimembranous VSD was most common type (67%) in our study. Interestingly, AVP is the precursor lesion in the pathogenic process of AR and was observed in 15 patients (20.8%) which is quite high as compared to its incidence in other studies by Lue et al.^8^ and Ando et al.^9^, thus pointing towards graver situation.

Furthermore, RCC was most commonly prolapsed (93%) and NCC was prolapsed in 6% of cases which is similar to findings reported in other local studies.^4^ Similarly, regarding incidence of perimembranous VSD, our results are in consonance with other local studies by Chudhry et al. and Kazmi et al. studies^10^,^11^ suggesting perimembranous VSD is the most prevalent lesion.^4^ The severity of aortic valve disease was also more in cases of perimembranous VSD than in DCSA VSD.

Severe pulmonary hypertension is well known complication of untreated VSD^10^. However, patients with AR and VSD usually do not have pulmonary hypertension because prolapsed aortic cusp limits the left to right shunt and keeps Qp/Qs ratio low. Similarly, it is quite understandable that pulmonary valve is protected in DCSA VSD as the valve is preserved by low pulmonary diastolic pressure while high systemic diastolic pressure pushes the aortic valve into the defect. Only one patient of our series has moderate pulmonary hypertension. Had it been the severe pulmonary hypertension, some of these patients would have become inoperable at the time they were referred to us.

Progression of AR was not observed even in a single patient with mild or trace AR as evidenced by results of Group I and II. Nonetheless moderate AR from Group-II (in one patient) and one patient from Group-IV did progress to severe AR. Likewise, Ishikawa et al. also showed that mild AR do not progress to moderate or severe AR, however moderate AR frequently progress to severe AR, so a careful surveillance is warranted in such cases.^12^

Karpawich et al. and Okita et al. reported that repair of perimembranous VSD defect along with AR is artily, as it involves both RCC and NCC, also, progression of repaired AR associated with perimembranous defect to severe degree is more frequent.^13^, ^14^ We also observed that not only the severity of aortic valve disease was more in perimembranous VSD but also progression of disease from moderate to severe AR occurred only in cases of perimembranous defects.

Surgical approaches for the aortic valve repair used in our study were Trusler repair, cusp augmentation, cusp perforation repair and new cusp formation with pericardium. Two patients underwent aortic valve replacement (19 mm mechanical valves St-Jude). Trusler repair popularized by Trusler and colleague^15^ is the most widely accepted method of repair that showed efficacy at long term surveillance of 10 years as shown by Trusler GA et al.^16^ and even at 15 years as shown by Elgamal et al.^17^ New cusp formation was invented by Bahnson et al.^18^ and later adopted by Henry et al. and modified by replacing Teflon with Glutaraldehyde fixed pericardium.^19^ They recommended it as only acceptable method for valve repair, in cases of severe aortic regurgitation that produces the long term good outcome.

Elgamal et al.^17^ after 15 years of follow up of post-operative patients of VSD and AR concluded few important risk factors for progression of AR after repair, which are, (1) degree of aortic insufficiency immediately after repair (2) direct closure of VSD and (3) usage of more than one plication sutures. Correspondingly, we also observed in our study that the two patients who progressed to severe AR had moderate AR following repair. We routinely close the VSD with a patch no matter how small it is.

Mean age of our study population was 12.93±6.75 years due to late diagnosis and inefficient referral pathway. Neither age nor gender was observed a risk factor for inadequate repair of AR and later progression of disease. Likewise, Trusler et al.^16^ after 10 years of follow up also showed lack of evidence for age as a risk factor. Albeit, Salih et al. showed female gender a risk factor for later progression of disease.^20^

Comparing our results to local studies, Kumari et al.^4^ showed 84% regression of aortic valve disease after surgical repair. They showed 57% of residual VSD which reduced to 30% after one year follow up. They also showed good prognosis for aortic valve repair at earlier age. Our study showed 94% regression of aortic valve disease and only one patient (5.8%), among those in whom we did aortic valve repair, and one patient (1.8%), among those in whom we did not do intervention, progressed to severe AR after follow up of 2.5 years. Only two patients (2.8%) showed residual VSD.

Once developed, AR associated with VSD is a progressive lesion with significant morbidity. However, this study showed that early treatment and effective follow up may regress and even halts the disease process. Delayed referral, lack of comprehensive follow up, and low socioeconomic strata are the reasons because of which it is imperative that government led effective guidance for early diagnosis, timely referral should be well in place to address the potentially avoidable morbidity associated with this condition.

### Limitations of the study:

The data presented is solely based on single surgeon and single center-based experience so the expertise from other surgeons and experience of other centers is required for validation of results. A small cohort and relatively short follow up periods are also considerable feeble points.

## CONCLUSION

Considering the unfortunately very high prevalence of congenital cardiac defects in our country this article provides an evidence of surgical results of our current clinical practice stressing the fact that aortic regurgitation associated with ventricular septal defect is a progressive condition associated with significant morbidity and mortality. Early management of aortic valvular disease slows and, in most cases, halts the progression of disease. Efficient and well-coordinated follow up pathway are imperative for post-operative surveillance.

### Author’s Contribution:

**TW:** Conceived, designed the research methodology, prepared this manuscript and is accountable for the originality of the research work.

**MFAR & JAN:** Did data analysis, helped in writing the manuscript and reviewed the manuscript.

**KK:** Acquisition of data and helped in writing the manuscript.
